# Active Ultrasound Pattern Injection System (AUSPIS) for Interventional Tool Guidance

**DOI:** 10.1371/journal.pone.0104262

**Published:** 2014-10-22

**Authors:** Xiaoyu Guo, Hyun-Jae Kang, Ralph Etienne-Cummings, Emad M. Boctor

**Affiliations:** 1 Dept. of Electrical and Computer Engineering, Johns Hopkins University, Baltimore, MD, United States of America; 2 Dept. of Computer Science, Johns Hopkins University, Baltimore, MD, United States of America; 3 Dept. of Radiology, Johns Hopkins University, Baltimore, MD, United States of America; University of Navarra, Spain

## Abstract

Accurate tool tracking is a crucial task that directly affects the safety and effectiveness of many interventional medical procedures. Compared to CT and MRI, ultrasound-based tool tracking has many advantages, including low cost, safety, mobility and ease of use. However, surgical tools are poorly visualized in conventional ultrasound images, thus preventing effective tool tracking and guidance. Existing tracking methods have not yet provided a solution that effectively solves the tool visualization and mid-plane localization accuracy problem and fully meets the clinical requirements. In this paper, we present an active ultrasound tracking and guiding system for interventional tools. The main principle of this system is to establish a bi-directional ultrasound communication between the interventional tool and US imaging machine within the tissue. This method enables the interventional tool to generate an active ultrasound field over the original imaging ultrasound signals. By controlling the timing and amplitude of the active ultrasound field, a virtual pattern can be directly injected into the US machine B mode display. In this work, we introduce the time and frequency modulation, mid-plane detection, and arbitrary pattern injection methods. The implementation of these methods further improves the target visualization and guiding accuracy, and expands the system application beyond simple tool tracking. We performed ex vitro and in vivo experiments, showing significant improvements of tool visualization and accurate localization using different US imaging platforms. An ultrasound image mid-plane detection accuracy of ±0.3 mm and a detectable tissue depth over 8.5 cm was achieved in the experiment. The system performance is tested under different configurations and system parameters. We also report the first experiment of arbitrary pattern injection to the B mode image and its application in accurate tool tracking.

## Introduction

Tool tracking/guidance techniques have broad utility in medical intervention and image-guided therapy. In medical interventions, including biopsy, ablative therapy, and cardiac catheterization, image guidance is the pivotal task and technology that determines operation success and guarantees clinical outcome. In literature, research shows that in many applications, intervention outcome can be greatly improved if an effective tool tracking method is introduced [1]. Various medical imaging modalities are used to recover the pose of the interventional tools [2]. Compared to CT and MRI, ultrasound (US) imaging has several advantages including low cost, safety, mobility and ease-of-use. Two major limitations, however, prevent conventional ultrasound imaging systems from providing effective tracking and guidance. The first one is the poor visualization of interventional tools. Ultrasonography reconstructs images from received echo ultrasound signals. In interventional procedures, many catheters or needles cannot be reliably visualized in ultrasound images due to the low echo signal amplitude, large reflection angle, strong tissue attenuation or the complicated image background [3]. The second limitation is the low localization accuracy in the elevational dimension. The acoustic beam width usually ranges from several millimeters to one centimeter, which means an object shown on a 2D ultrasound image has an inherent uncertainly of a few millimeters to one centimeter along the elevation axis.

In recent years, several approaches have been proposed to enable tool visualization and pose recovery, including beam steering, optical tracking, electromagnetic (EM) tracking, and passive/active ultrasound markers [4]–[11]. The beam steering method has been developed and validated by several research groups and currently is integrated into a commercial ultrasound scanner from SonoSite Inc. The basic principle is to steer the imaging beam directions to get the optimized reflection from the catheter. It has been proven effective when the catheter is in-plane of the US image. For cases where catheters simply intersect the imaging plane with a large angle, this method is not effective [4]. In the optical tracking approach, an optical marker is attached at the end of the tool and uses a stereo camera to capture the 3D position of the marker [5]. This approach suffers from several limitations, including high cost, calibration complexity, intrusive setup, needle bending error, and the need for clear line-of-sight from the camera to the tool. Electro Magnetic (EM) tracking approach was recently introduced and integrated into several commercial ultrasound scanners (GE LOGIQ E9, Ultrasonix GPS, etc.). In this approach, an EM field is generated by an EM emitter; EM sensors are implanted in both the catheter and the imaging probe. The relative pose of the catheter is estimated and injected as a graphic overlay to the B-mode image [6]. The EM tracking quickly became a commercialized technology due to its merits of excellence in off-plane detection capability. However, several disadvantages limit its applications to all interventions: the overall navigation accuracy can easily be worse than 2∼3 mm; it requires specially designed imaging equipment; and any ferromagnetic object in the operation region may affect the system accuracy. Another approach with a similar concept is to use ultrasound sensors instead of EM sensors. Several research groups have demonstrated the system in which an ultrasound receiver is attached to a catheter. The catheter localization is retrieved by measuring the timing of the ultrasound pulses from the imaging probe [8]–[10]. This method requires specialized US system and image processing software; thus its application is limited.

Other than detecting the tool spatial location, some researchers focus on the visualization enhancement of the catheter in B-mode images. One approach is to improve image quality by using passive ultrasound markers. In this method, ultrasound markers, sometimes in the form of scattering coatings, are integrated into the tool to improve the echo amplitude [7]. The major issue of this approach is that the visualization enhancement by scattering coating is limited; with markers, they are usually too bulky for small catheters. On the other hand, active ultrasound marker approach has also been proposed. F. Simonetti introduced the concept that using biopsy needles as ultrasound waveguide [11]. An ultrasound wave is injected from the end of the needle. It travels along the needle and leaks into the tissue, and is picked up by an external imaging probe working in passive mode. The limitation of this method is that the phase, waveform, timing and leaking position of the guided beam cannot be well controlled; It requires the ultrasound system to stop normal operation and work in passive mode, which is not a direct visualization enhancement; its performance highly relies on the mechanical property of the needle and cannot be generally applied to any surgery tools. B. Breyer and I. Cikes reported an active ultrasound marker system that fires an ultrasound signal from the catheter, which shows a visible pattern in the B-mode image [12]. The timing is acquired by receiving the imaging pulses from the ultrasound probe. This approach directly injects the marker in the B-mode image and does not require special designed imaging system. B. Breyer's and I. Cikes' work for the first time presented the active marker concept on the medical ultrasound imaging system. However, this method was not eventually used in clinics. Experiment results show that in the in-vivo B mode images, it is difficult to identify the active echo spot from the tissue texture. Even in the water tank environment, a simple receive and echo configuration does not provide enough feedback to the operator; the echo spot may show up within a large range of tool position, the elevation localization accuracy is poor.

In this paper we introduce a new technique called ultrasound active pattern injection, a further development of the active visualization enhancement approach. Unlike previous works, the tool visualization and localization is not simply achieved by introducing an ultrasound beam to the tissue or echoing the imaging pulses. The Active Ultrasound Pattern Injection System (AUSPIS) is composed of an interventional tool with one or multiple ultrasound elements (active echo element), ultrasound analog frontends, a signal processing system, and a pulser. The system receives the image beacon pulses, analyzes the acquired signal, and fires one or a series of active echo pulses from the same active echo element with a proper timing, frequency, duration and amplitude. Thus it enables us to inject any “virtual” pattern into the B-mode image. Unlike the image overlay technologies used in other tracking or image registrations, the pattern injected to the B mode image from the actively encoded ultrasound field in the tissue. The encoding is based on the B-mode ultrasound image forming mechanism, so it doesn't require any hardware or software modification to the ultrasound machine. It continuously measures the local acoustic signal amplitude, by which a sub-millimeter elevation localization accuracy can be achieved. By configuring the pattern formations, the technique can be used for tracking, tool guiding, and annotation. In this study, we 1) developed a hardware and firmware prototype of the AUSPIS; 2) performed the lab bench experiment to test the design idea and investigated the working conditions and limitations of the system; 3) tested the system performance in-vivo.

## Methods

**Figure 1:**
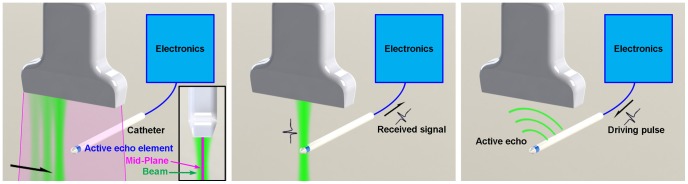
The configuration of the active reflector tracking system. Left: the imaging probe fires a serious of ultrasound beams scanning the image region. The center plane of the image region is the mid-plane. Middle: When an ultrasound beam intersects the active echo element, an electrical pulse is received by the electronics. Right: The received pulse triggers the electronics, a driving pulse is sent to the element to fire an active echo pulse.

The basic principle of AUSPIS is shown in figure 1. When acquiring a B-mode image, one or a group of array elements is driven to fires an ultrasound pulse, which propagates along a transmission beam line. After the pulsing, the array is switched to receive mode to acquire the echo. Element groups are fired one by one until the entire image region is covered to reconstruct ultrasound Brightness mode (B-mode) from the multiple RF lines. In the active pattern injection system, a small ultrasound transducer (active echo element or AE element) is integrated with the catheter. When a transmission beam reaches the element, an electrical pulse signal is excited and sent to the electronics. To simply improve the tool visualization, the electronics drive the AE element immediately to send an ultrasound pulse back to the imaging probe. The receiving-transmitting delay is in nanoseconds and negligible for US imaging. The active ultrasound pulse is superimposed on the catheter echo wave, resulting in an enhanced echo pulse with a much higher amplitude, broader frequency range and wider emission angle. This pulse travels back to the imaging probe, and appears as a bright spot (AE spot) that indicates the AE element location in the B-mode image.

As described previously, simply echoing the imaging pulses is not very useful in many cases. However, based on the signal received by the AE element, more complex functions can be developed to improve the tracking performance.

### 1. Time modulation

In clinical or ex-vivo conditions, simple active echoing has very limited improvement in the visualization, because a static echo spot can barely be seen from a complex B-mode background. To overcome this problem, the signal processing unit periodically enables/disables the active echo feedback. As a result, the active echo spot blinks in the B mode image with the same period, making it distinct from the background B mode textures. The tissue phantom and in-vivo experiments proved a good improvement of catheter visualization and tracking using this method.

### 2. Frequency modulation

The signal processing unit can also control the echo pulse frequency and duration. Although the signal waveform is not shown in the B-mode image, in the raw ultrasound or beam-formed RF signals, filtering method can be used to differentiate the background B-mode signal and the active echo signal, which has a different frequency or waveform characters than the imaging pulses. This can be used for the automatic tool guidance.

### 3. Mid-plane detection

In surgical interventions, even if the tool shows good visualization in B-mode image, it is difficult to determine whether it is well aligned with the image mid-plane due to the ultrasound beam width, as was previously described. To achieve high localization accuracy, a tracking system should be able to detect the image mid-plane, as shown in figure 1. In ultrasound imaging, the beam transmitted from an imaging probe has higher power intensity at the center, and lower intensity away from the center. Since the AE element is capable of measuring the local ultrasound pressure in real time, the beam intensity distribution can be utilized to localize the mid-plane. In the AUSPIS, when the AE element is well aligned with the mid-plane, the received beacon signal amplitude reaches its maximum, and when it moves away, the strength of beacon is decreased. Practically, the US beam intensity is determined by many factors including probe type, tissue attenuation, image depth, focusing point, etc. It is difficult to preset a trigger threshold that makes the AUSPIS only respond to the maximum US signal. However, we found that if the threshold is set to a relatively low value, the AUSPIS will be triggered multiple times in each image frame, and the trigger count, which is defined as the number of triggers received in each frame, reaches its maximum when the AE element is well aligned with the mid-plane. This is because when the US probe scans the FOV, each beacon beam has spatial overlapping with one or more of its neighboring beams. So the AE element receives US signal not only from the beam that intersects it, but also from the nearby beams, just with lower amplitude. When the AE element moves closer to the mid-plane, the received signal amplitude increases; as a result, more nearby beams exceed the threshold, so the trigger count increases. Since the trigger count indicates the alignment between AE element and mid-plane, it can be used for mid-plane detection. AUSPIS is able to read the trigger count and feedback to the operator in multiple ways, like a number displayed on the screen, a beeping sound with a frequency proportional to the trigger count, or an injected virtual pattern on the B-mode image, which will be discussed later.

### 4. Arbitrary pattern injection

**Figure 2:**
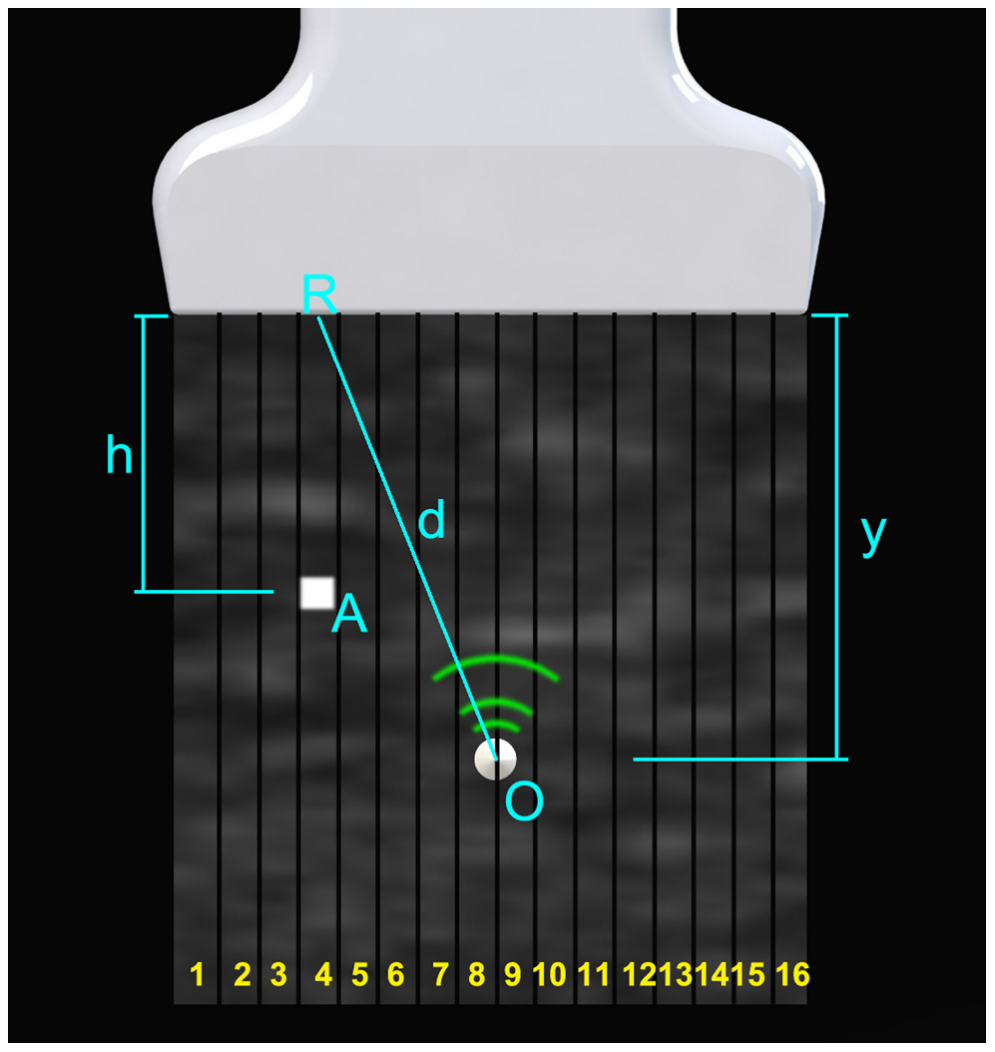
B-mode Pattern injection with a linear array. This picture illustrates how a single virtual pixel is injected to the B-mode image from an active echo element.

The pattern injected into the image is not limited to the AE spot. As described previously, a B-mode image is formed by a series of A-mode lines. As shown in figure 2, suppose a B mode image is composed of 16 A-mode lines; the normal distance from the AE element to the probe is *y*. To generate a virtual spot on the point *A*, an ultrasound pulse should be received by the probe when the A-mode line #4 is being acquired, with a delay of
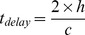
where *c* is the speed of sound in this medium. The ultrasound pulse is generated from the AE element at position *O*, the distance between *O* and the center of the imaging elements *R* is *d*. The time for sound to travel from *O* to *R* is *t_travel_ = d/c*. So the timing that the element should send an ultrasound pulse is:
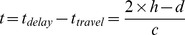
In other words, if an ultrasound pulse is fired from the AE element *t* seconds after the probe starts acquiring the *A* mode line #4, it will be shown as a virtual spot at position *A* in the B-mode image.

Having these spots as “pixels”, arbitrary patterns can be formed and injected to the image. The challenging issue is that the relative position between *O* and *R* is unknown in the real application scenario. Two methods can be used to overcome this problem. The first method can be used if the ultrasound system is able to output synchronization signals. Since the linear array size, the total number of A-mode lines, and the speed of sound are known parameters, *d* can be automatically determined from the delay between received signal and the synchronization signal. In this case the arbitrary pattern can be injected into any pre-defined position in the B-mode image, i.e., the injected pattern can be either at an absolute position in the image coordinate, or at a relevant position moving with the active element. The second method can be used when the synchronization signal is unavailable. In this case injecting a pattern to an absolute position in the image coordinate is not possible. However, since the AE element is able to receive multiple neighboring beacon pulses when the probe is acquiring the nearby A-lines, the A-line period can be acquired by the system. Using the received pulse as the synchronization signal, and calculating the desired time delay from the A-line period, virtual patterns can be injected into the B-mode image with the AE element coordinate. The implementations of both methods will be presented in the following paragraphs.

## Prototype System

**Figure 3:**
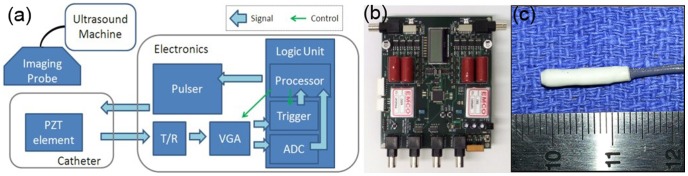
The prototype AUSPIS setup. a) The block diagram of the AUSPIS. b) The pulser and logic unit board. c) One of the prototype catheters; it has an active echo element very close to the tip.

Figure 3a shows the block diagram of the AUSPIS prototype. A piezoelectric element for both ultrasound receiving and transmitting is integrated with a catheter. The element is connected to a customized electronic circuit, which consists of a transmit/receive (T/R) switch, a variable gain amplifier (VGA) with analog filters, triggering circuit, analog to digital converter (ADC), an embedded microprocessor, and a pulser. Figure 3b and 3c show the circuit board and the catheter tip. The T/R switch circuit is built with a TX810 8 channel integrated switch to protect the receiving circuit from the transmission driving voltage, which can go up to a hundred volts. Analog filters have a low and high cutoff frequency of 0.1 MHz and 20 MHz. The trigger circuit compares the absolute value of the signal amplitude and the threshold, and sends pulses out when it is higher than the threshold. Since the receiver and emitter are the same element, the trigger circuit has a latch and reset function to prevent oscillating triggering. The ADC converts the analog signal to digital. The control program runs on an embedded processor, which controls all the peripherals on the AUSPIS circuit. To drive the element, the pulser is able to generate pulses with a minimum duration of 12.5 ns and variable voltage from zero to ±150 V. The active echo element is a customized small tube made of PZT5-H material with an outer diameter of 2.08 mm, inner diameter of 1.47 mm, and length of 2 mm. The catheter has a sealing layer around the element, which makes the overall diameter of the catheter tip close to 3.0 mm. A Sonix RP and a Sonix Touch ultrasound systems (UltraSonix Co.) with L14-5W (128 element) linear probe and 4DL14-5/38 (128 element) 3D probe is used in the experiment. We've also tested the AUSPIS with SonoSite portable ultrasound system to verify the cross platform operation performance.

## Results

### 1. Active echo validation

**Figure 4:**
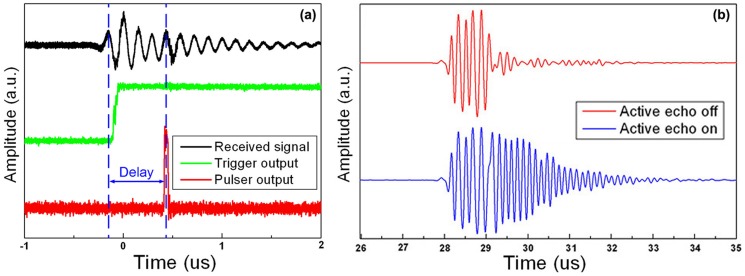
The system received and transmitted signals. a) The signal received by the active echo element (black), the output of the trigger unit (green), the output of the active echo driving pulse (red). b) A RF line received by the imaging probe with and without enabling the active echo system. The active echo adds a ringing tail after the original signal.

Figure 4a indicates that the prototype device has an overall electronic loop delay of 0.6 µs. Due to this delay, the active echo spot is about 0.3 mm away along the axial direction from the true element position in the B-mode image. Figure 4b shows the signal received by the imaging probe. The red waveform is the reflection from the catheter; the active echo system is not enabled. The blue line shows the received waveform when the active echo is enabled. From this plot we can clearly see that the active echo is fired after the reflection signal, with a delay of around 1 µs. The additional 0.4 µs delay may be due to the response of the prototype PZT element, which is relatively large in size. The active echo adds a ringing tail after the original signal. The frequency of the active echo is adjustable; in this plot, it has a slightly higher frequency than the original signal.

### 2. Frequency modulation

**Figure 5:**
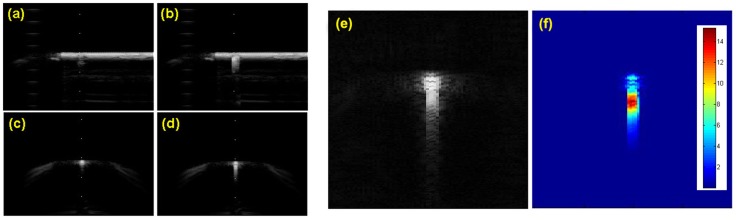
The active echo image in water tank. The catheter used in this experiment has an AE element integrated about 5 mm away from the tip. a)∼d) The B-mode image of the catheter in water tank. a, b) the in-plane configuration, c, d) the off-plane configuration. The active echo is enabled in b) and d). e) The B mode image of the catheter with the active echo signal. f) The active echo signal extracted from the B mode image using the template filtering method. The color code represents the convolution value between the signal and template.

Figure 5 a)–d) shows the B-mode image with and without enabling the active echo. Due to the transmission signal ringing after the main pulse peak, the active echo spot has a tail away from the US probe. Longer ringing tail improves the active echo spot visualization; at the same time it may decrease the visual localization accuracy in some cases. An adjustable termination circuit can be used to control the ringing settling time. Figure 5 e) and f) shows the original active echo B-mode image and the result after template filtering. The waveform template of the active echo is acquired by subtracting the RF lines with and without active echo enabled.

### 3. Mid-plane detection

As discussed previously, either the peak signal amplitude or the trigger count can be used in mid-plane detection. Practically, in the trigger count method the AUSPIS starts to response the beacon pulse even if the AE element is not well aligned with the mid-plane, it provides a large detection range, which is useful for a quick target search before the accurate tool tip localization. Once the active echo signal is received, the peak signal amplitude method can be used for a more accurate alignment.

**Figure 6:**
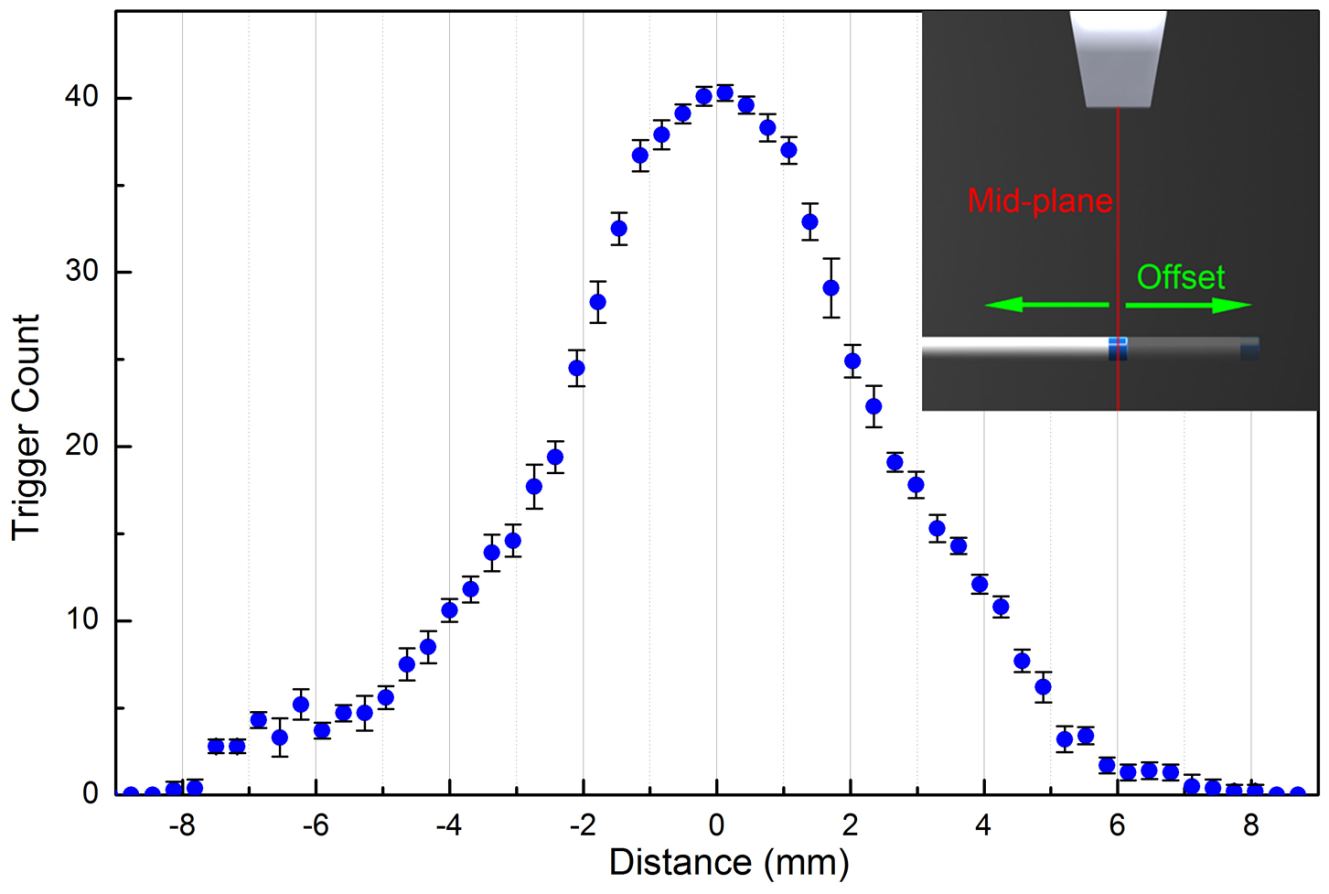
The trigger count versus the offset between the active element and the mid-plane. In this experiment the catheter is fixed in a water tank and perpendicular to the image plane. The probe is mounted on a translation stage, which moves perpendicular to the mid-plane. The error bar represents the standard deviation over 10 measurements, each time the translation stage moves from −9 mm to 9 mm, stops at the same sample positions and takes the trigger count reading.

A typical trigger count versus AE element to mid-plane distance plot is shown in Figure 6. The data is acquired with a fixed AUSPIS receiver gain of 19 dB. The AE element is about 4 cm away from the probe. The US probe is running on its maximum transmission power with 32 element transmission aperture and 4 cm focus depth. In each frame the probe fires 256 RF lines. When the offset increases, the frame trigger count drops from the maximum 41 to 0 at an offset of 8 mm. The result indicates that, with these system parameter settings, the active echo detectable range is about ±8 mm from the mid-plane. The range can be increased or decreased by using higher or lower amplifier gain.

**Figure 7:**
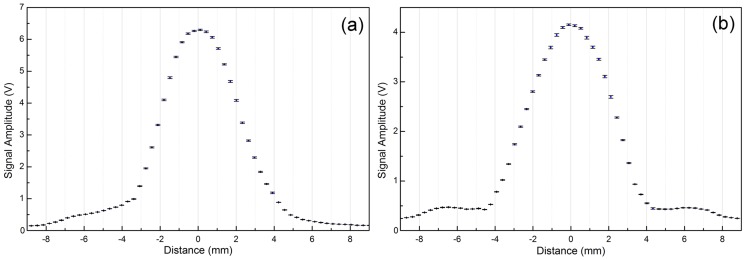
The local ultrasound amplitude measurement for mid-plane detection. a, b) the amplitude of the signal received by the active echo element verses the position of the probe. The depth of the US transmission beam focus is close to the catheter to probe distance, so the two plots are showing the signal distribution near the focus. a) The results with catheter perpendicular to the image plane (off-plane) b) catheter parallel to the image plane (in-plane). The error bar represents the standard deviation over 10 measurements, each time the translation stage moves from −9 mm to 9 mm, stops at the same sample positions and takes the signal amplitude reading.

Once the AE element is triggered, the next step is to accurately align it to the mid-plane. Figure 7a, b shows the catheter received signal amplitude verses the imaging probe position. In the plot, the mid-plane position is indicated by the maximum of the signal amplitude. If the trigger level is set to a value close to the maximum, the active echo system can only be triggered when the element overlaps the mid-plane. For example, if a trigger is set to 90% of the maximum amplitude, a localizing accuracy of ±0.5 mm is expected from the Figure 7. In the water tank validation, the receiver gain is decreased until only one trigger per frame is achieved at the well aligned position. Then gradually move the catheter away from the mid-plane. At an offset of ±0.31 mm, the active echo spot disappears. The result is repeatable and stable over test runs. Using this method, a mid-plane localization accuracy of 0.3 mm can be achieved.

### 4. Insertion angle test

In the previous tests, the catheter is perpendicular to the imaging ultrasound beams. However, this condition is not always satisfied in clinical applications. An experiment is performed to validate the system performance when the catheter is inserted at an angle. Since the prototype active element has a cylindrical shape, the most sensitive configuration is the cylinder axis perpendicular to the ultrasound beam, which is marked as 0° in the experiment. Keeping the element at the same location, the catheter rotates from −50° to 90° with 10° increment steps. Because the element is not an omnidirectional sensor, the received ultrasound pulse amplitude varies with the angle, so the receiver gain needs to be adjusted at different angles. The active echo performs properly at all angles we tested in the experiment. Because the catheter interferes with the ultrasound probe, we were not able to test at angles from −90 to −50 degrees.

### 5. Validation with different ultrasound powers, apertures, focuses, orientations, and gains

**Figure 8:**
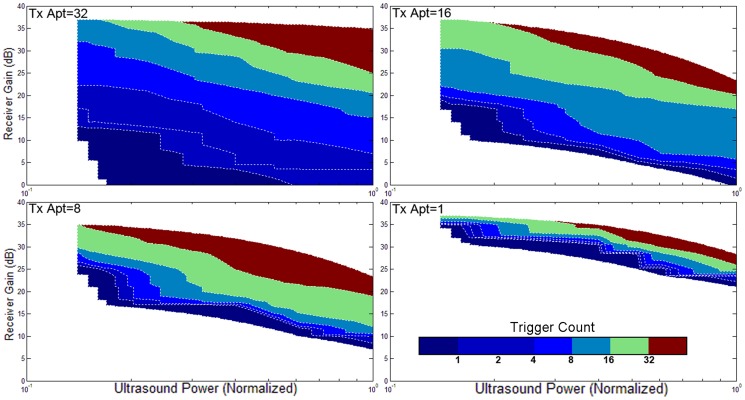
The trigger count versus ultrasound system settings. Catheter is perpendicular to the image plane. Ultrasound beams are focused to the catheter insertion depth.

**Figure 9:**
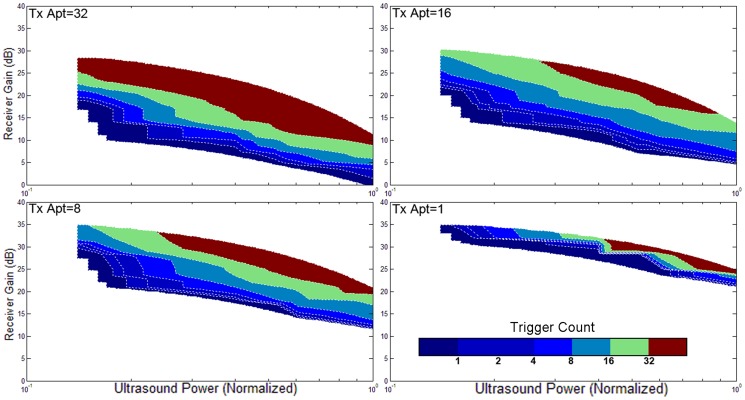
The trigger count versus ultrasound system settings. Catheter is perpendicular to the image plane. Ultrasound pulses are not focused.

**Figure 10:**
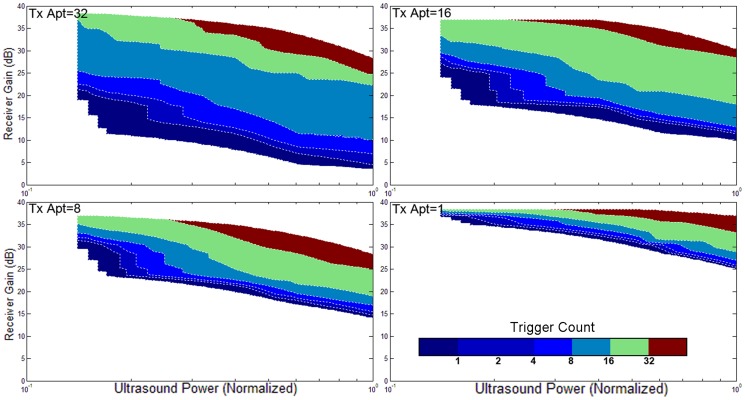
The trigger count versus ultrasound system settings. Catheter is parallel to the image plane. Ultrasound beams are focused to the catheter insertion depth.

**Figure 11:**
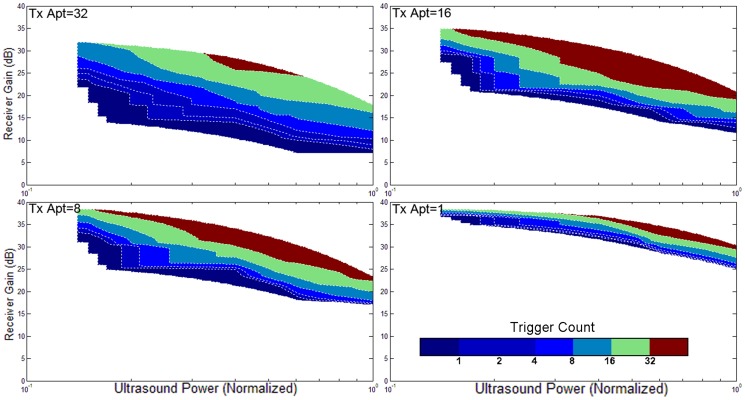
The trigger count versus ultrasound system settings. Catheter is parallel to the image plane. Ultrasound beams are not focused.

The triggering condition of the active echo system is mainly affected by two factors: the amount of acoustic power received by the active element, and the gain of the receiver. The former factor also relies on many parameters like the probe transmission power, transmission aperture, focusing, tissue attenuation, etc. An experiment is performed to investigate the working conditions of AUSPIS. In this experiment, the active echo element is aligned with the probe mid-plane in a water tank. An attenuation layer is placed between the probe and catheter to mimic tissue attenuation. The trigger count is recorded under different parameter settings. Shown in Figure 8–11, the color code indicates the trigger count per frame. Generally, to achieve the same trigger count, it requires more probe transmission power at lower receiver gain than at a higher gain. Larger transmission aperture delivers more acoustic power, thus resulting in a higher trigger count when other parameters are the same. Comparing the focusing and no focusing, the former one is relatively less sensitive to the receiver gain setting, i.e., the trigger count changes less when varying the gain. Take the figure 8 and 9 “Tx aperture = 32” as an example, the color bands between the contour lines are wider in 8a, which indicates that, within a larger range of receiver gain setting, the trigger count remains the same. This is because the focused beam has smaller beam width, when the ultrasound probe acquires a B-mode image frame, only the A-mode lines close to the element can trigger the active echo. Since the energy is spatially concentrated, less transmission power is required to trigger the active echo, so in the focused beam conditions, the functional zone (colored area) extends to lower gain area, as shown in figure 8. In the unfocused case, since the beams are wider and have more overlapping, the beams far away from the element may also trigger the echo, so the trigger count growths rapidly when increasing the gain. Similarly, smaller transmission aperture results in wider beam, the bands are narrower than that with the larger aperture. In the experiment, the data with a trigger count higher than 40 are discarded; that is why the colored area does not extend to the very high gain region in figure 8–11. Though it may be useful in the quick-tool-searching purpose, high gain with large trigger counts makes the active echo spot wide and distorted, which may result in an inaccurate tool indication in the original B-mode image.

### 6. Detection depth test

**Figure 12:**
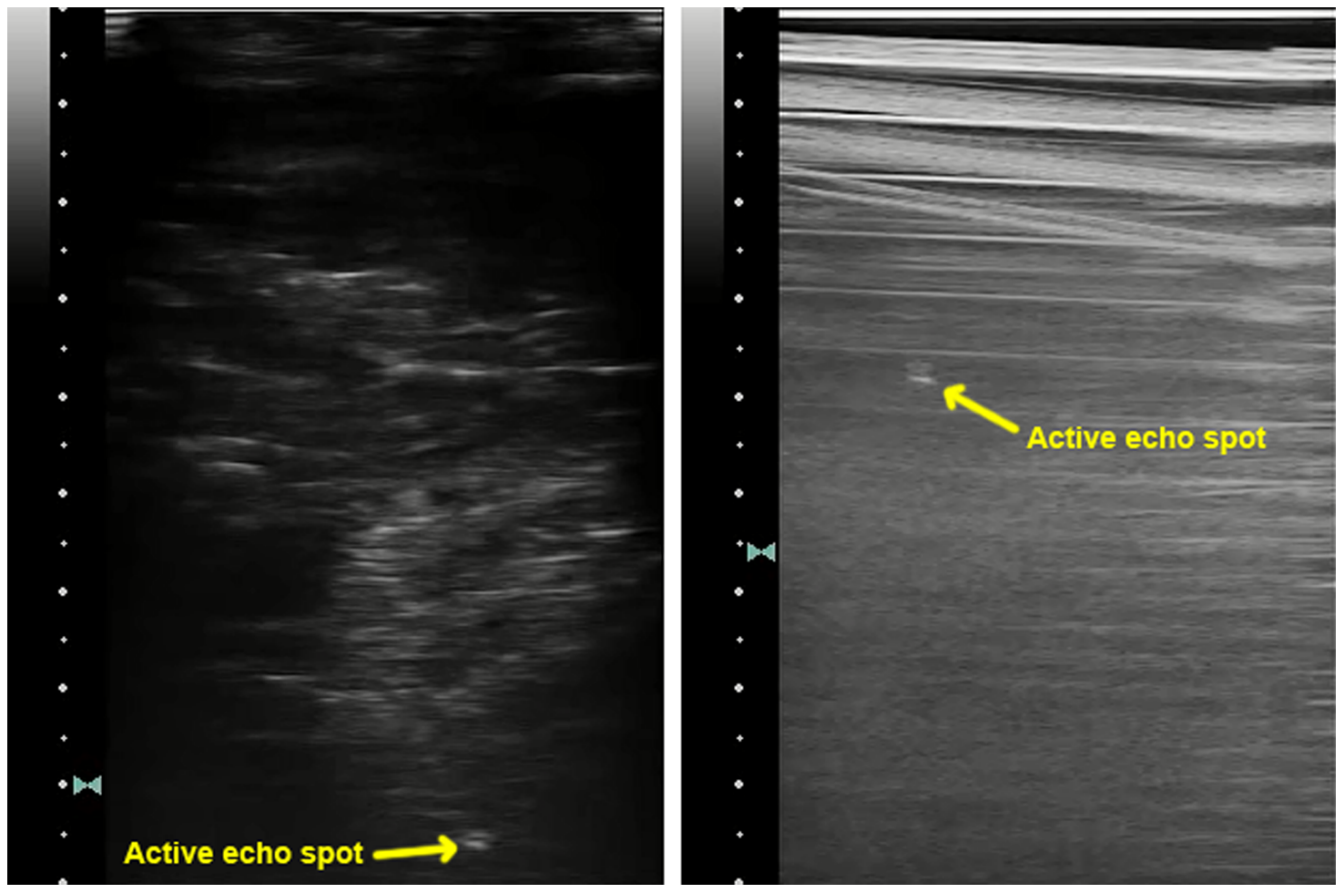
Large depth and impedance mismatching condition test result. Left: the active echo spot under 8.5 cm deep chicken breast tissue. Right: the active echo spot below a 1 inch thick aluminum plate.

To further investigate the extreme functional conditions of the system, we performed two lab bench experiments. In the first one, we insert the catheter into ex vivo chicken breast tissue at 8.5 cm depth, which is close to the limit of the probe we use. This is to mimic the deep needle insertion in clinical operations. An ultrasound video is given in Video S1. In the second experiment, we placed a 1 inch thick aluminum plate in the water tank between the imaging probe and catheter to mimic the impedance of highly mismatched tissues like hard bone. As shown in figure 12, in both of the two experiments, the catheter can be effectively localized by the active echo spot. In the second test, since the speed of sound in the aluminum layer is much higher than that in water, the spot looks much closer than it actually is in the B-mode image.

### 7. Validation with different imaging modes

Most clinical ultrasound systems provide different imaging modes, like harmonic mode (or pulse inversion mode), high resolution mode, high penetration mode, etc., which also affect active echo system performance. Using the Sonix Touch system with a L14-5W linear probe, we tested the active echo under four modes: harmonic mode, general mode, resolution mode, and penetration mode. Videos of the ultrasound system display are given in Videos S2, S3, S4, and S5. In Harmonic mode, the ultrasound system transmits pulses with central frequency of 5 MHz, and reconstructs the B-mode image from the received second harmonic (10 MHz) signals. In both general and resolution mode, the transmission frequency is 10 MHz; the difference is that in the resolution mode, the Rx frequency band is slightly higher than the general mode for better image resolution. In the penetration mode, the Tx frequency is reduced to 6.6 MHz to improve the penetration depth. We compared the signal to noise ratio (SNR) and contrast to noise ratio (CNR) of chicken breast tissue images using these imaging methods. The SNR is defined as:
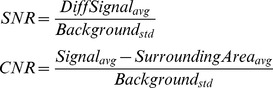
where the “*DiffSignal*” is differential signal extracted by using the echo on frame subtracting the echo off frame, the “*background*” is the echo off frame, the “*SurroundingArea*” is a 50 by 50 pixel area around the active echo spot.

**Figure 13:**
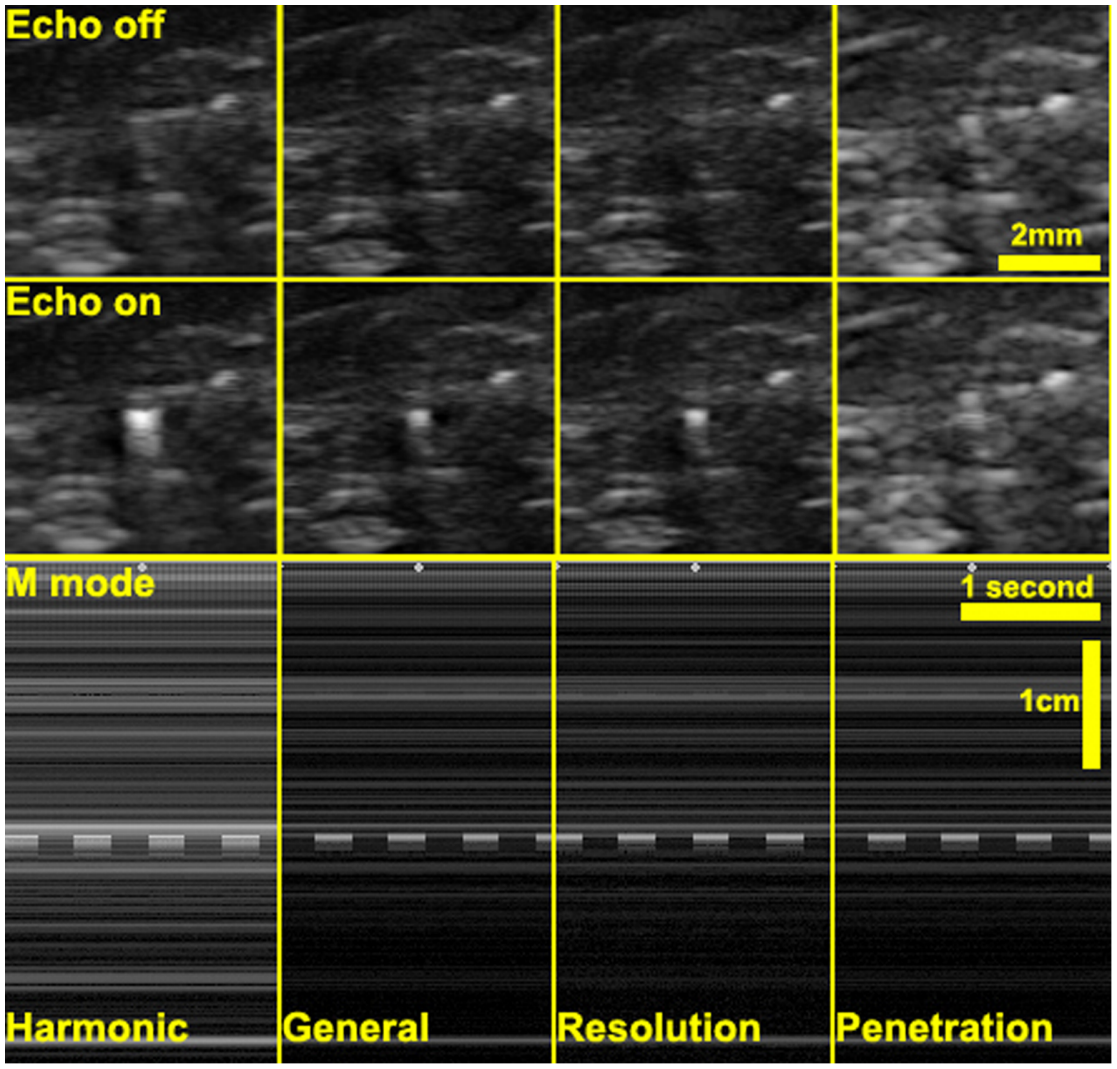
The B-mode and M-mode image of active echo spot using different imaging methods. The echo spot blinking can be clearly seen as a “dashed line” in the M-mode image. The blinking frequency is about 2 Hz.

Comparing the B-mode images in figure 13, the harmonic-mode image has the most observable echo spot. This is expected since the harmonic imaging relies on the tissue harmonic echoes, the amplitude of which is much lower than that of the original pulses. The active echo element is configured to transmit at a frequency close to the second harmonic (10 MHz) in this experiment, so the active echo signal significantly stands out from the tissue harmonic background. The penetration-mode image has the least observable echo spot, in part because the active echo frequency (about 9 MHz) is out of the Rx frequency band in this mode. This observation is consistent with the SNR and CNR measurements, as shown in table 1. However, even in the penetration mode, though the active echo spot is not clear in the static images, it is easy for the human eye to capture the spot during the operation due to its blinking.

**Table 1:** The SNR and CNR under different ultrasound imaging mode.

	Harmonic	General	Resolution	Penetration
CNR	5.93	4.65	5.10	1.92
SNR	5.66	4.35	4.77	1.78

### 8. Validation with different AE transmission powers

**Figure 14:**
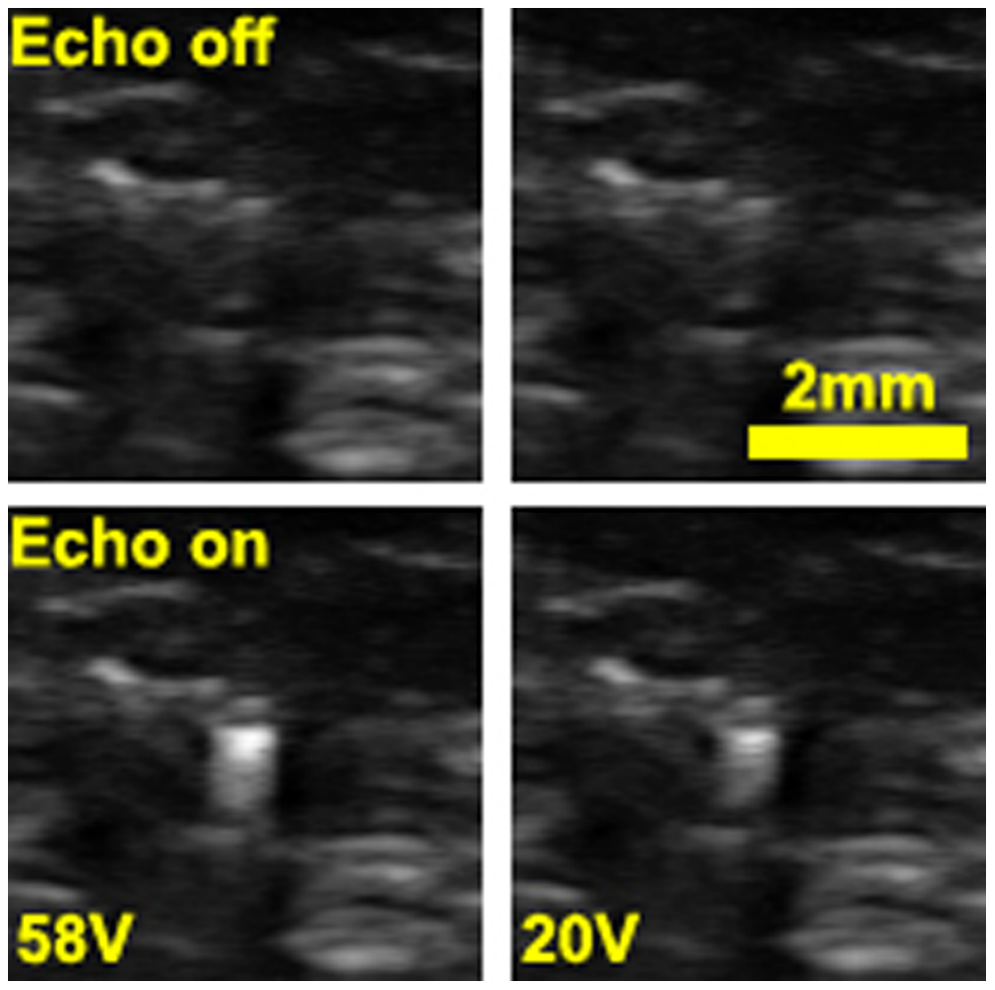
The active echo spot with different driving voltage. The left images are the active echo spot and the reference frame at 58 V driven voltage. In the right images, the driven voltage is reduced to 20 V. The echo intensity is dimmer on the right image; a lower SNR and CNR is expected.

Despite the imaging methods, the driving voltage of the element also affects the active echo spot visualization, since higher driving voltage results in stronger ultrasound. As shown in figure 14, with 20 V driving pulse, the echo spot is dimmer than that with 58 V driving pulse. However, since the B-mode image does not linearly represent the signal strength, we could not see the linear relationship in the CNR and SNR measurements, as shown in table 2.

**Table 2:** The SNR and CNR with different driving pulse voltage.

	Pulse peak voltage = 58 V	Pulse peak voltage = 20 V
CNR	6.20	4.73
SNR	6.36	4.51

### 9. Arbitrary pattern Injection

**Figure 15:**
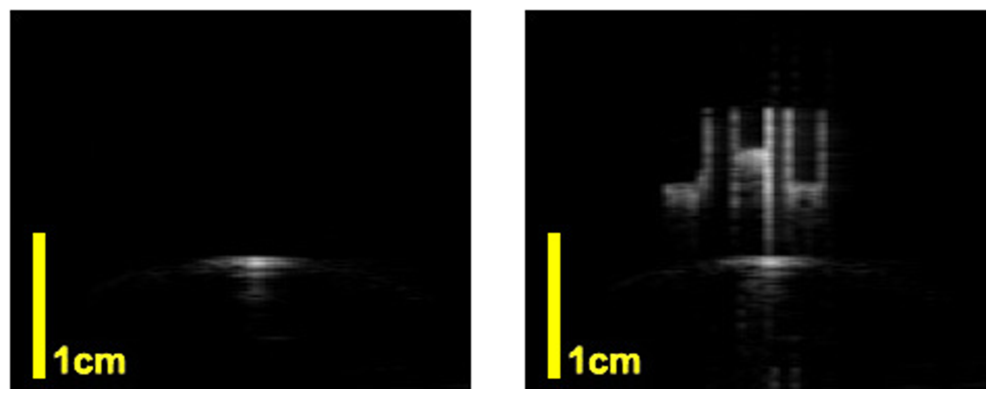
The B mode image of arbitrary pattern injection. The experiment is in a water tank with Sonix CEP machine and L12-5 linear probe. Left image is the reference without turning on the AUSPIS. On the right image, a virtual “JHU” pattern is injected to the image.

Figure 15 shows an example of arbitrary pattern injection method. The left image is the reference, the B-mode image of the catheter in a water tank, and right is the image with the pattern injection system turned on. A virtual “JHU” pattern is shown in the B-mode image. A video is given in Video S6. In this image, the “pixel” that forms the pattern can be seen. Each pixel corresponds to one ultrasound pulse firing from the catheter. Method 1 mentioned in the pattern injection paragraph is used in this experiment. Ultrasound system synchronization trigger is utilized, and the pattern is injected into an absolute position in the image coordinate.

### 10. Mid-plane indication using pattern injection

**Figure 16:**
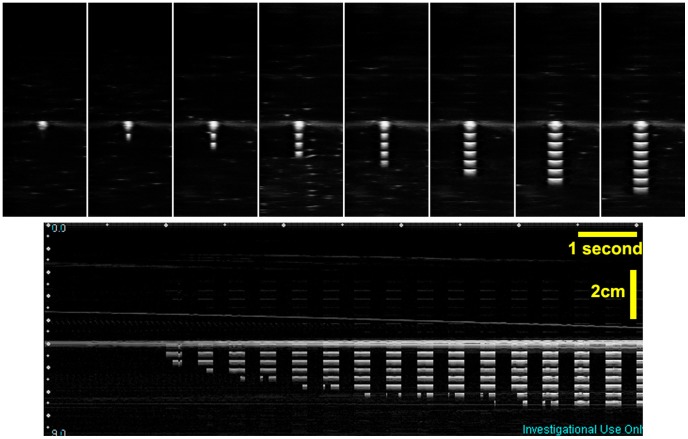
Mid-plane indication using the pattern injection method. The top column shows when the catheter moves closer to the mid-pane, the number of injected virtual bar increases. More bars means higher trigger count, thus indicating the catheter is closer to the mid-plane. The bottom picture shows an M-mode image acquired during the catheter moving in process.

In the previous paragraph, we introduced the method to detect the image mid-plane. The detection result can be directly displayed on the B-mode image using arbitrary pattern injection technique. As shown in figure 16, virtual bars that are proportional to the trigger count are injected into the image. With this real-time feedback, the operator can easily navigate the tool tip to the ultrasound image mid-plane, which is indicated by the maximum number of bars. Videos of this experiment are given in Videos S7, S8. This experiment is an implementation of method 2 mentioned in the pattern injection paragraph. Only the received signal is used for synchronization, and the pattern is injected into a relevant position moving with the active element in the image.

### 11. In vivo experiment

**Figure 17:**
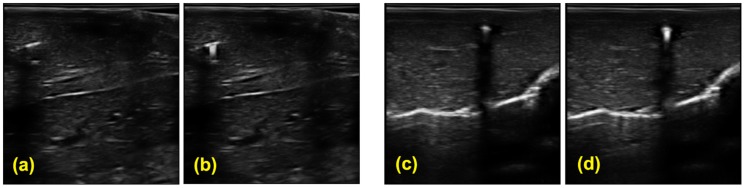
In vivo B-mode images extracted from the 3D volumetric data. The imaging array scans perpendicularly to the image plane with a step size of 0.5 mm. a–b) B mode images with the catheter in-plane. c–d) B mode images with the catheter perpendicular to the image plane. a) & c) Reference images with active element turned off. b) & d) images with the active echo spot.

The in vivo experiment was performed on a pig. The catheter is inserted into the liver tissue. 3D Volumetric B-mode images are collected by a 3D wobbler probe 4DL14-5/38. Figure 17 shows the images of the liver tissue with the catheter inserted. In a) and b), the catheter incidents the B-mode image plane with a small angle. c) and d) has the catheter perpendicular with the image plane. Although the catheter is visible in these B-mode images due to its large diameter and small insertion angle, it is not very distinguishable from the tissue texture, as shown in figure 17 a). Also, it is difficult to identify the location of the element, especially in the condition of c). In figure 17 b) and d), the active echo is enabled. With the echo spot clearly shown in the image, it is easy to identify the catheter from the tissue, and locate the element position.

## Discussion

The previous discussions are based on linear imaging arrays. For phased arrays, theoretically the active reflection, blinking and frequency modulation functions should have similar performance. The arbitrary pattern injection, however, works differently in the case of phased arrays or curved linear arrays due to the different image forming methods and parameters. How the system works with different type of probes is a future work to be investigated.

**Figure 18:**
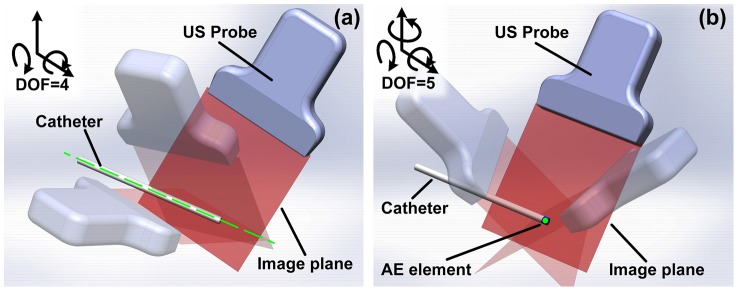
Degree of freedom (DOF) comparison between AUSPIS and beam steering method. a) Beam steering method requires the image plane to intersect the catheter line (the green line in figure a), the probe DOF is 4. b) The AUSPIS requires the image plane to intersect the AE element point (the green dot in figure b), the probe DOF is 5.

With single AE element, the AUSPIS provides 5 degrees of freedom to the probe, as shown in figure 18. It limits the image plane to intersect the AE element, which is a point. As a comparison, active beam-steering method constrains the image plane to intersect the catheter line, thus resulting in 4 degrees of freedom. In clinic, more DOFs mean less constraints on the probe position and orientation; this is especially important in applications like cardiac catheterization, in which doctors have to image from the very limited imaging window to avoid the ribs.

Like any other tracking system, AUSPIS has its limitations. This method is not able to localize the tool or project the insertion path when the AE element is far away from the ultrasound image plane. Using catheters with single AE element, the maximum detectable range in our experiments is around ±12 mm from the mid-plane. However, for certain applications, it can be improved by integrating multiple AE elements at different locations of the tool. Moreover, using multiple active elements not only the tool position but also the pose can be tracked. Another limitation in the prototype system is the shape and size of the AE element. For easy assembling and wiring, the piezoelectric element we use is a 2×2 mm cylindrical tube. The localization accuracy is expected to improve by reducing the element size. Last but not least, the operation of AUSPIS is very different from existing tracking methods. Although we have demonstrated the concept in both ex vivo and in vivo experiments, further study is needed to investigate how the technology can be integrated into the clinic workflow.

## Conclusions

In summary, we introduced a new active ultrasound pattern injection method for interventional tool tracking, designed and built a prototype system, and performed preliminary ex vivo and in vivo experiment to prove feasibility. Significant visualization enhancement and highly accurate mid-plane localization have been achieved. Arbitrary pattern injection has been demonstrated. We also presented the study and analysis of the system operating under different configurations. Finally the limitations and future developments have been discussed.

## Supporting Information

Video S1 The active echo spot under 8.5 cm deep chicken breast tissue.
(AVI)

Video S2 The active echo spot in the harmonic mode.
(AVI)

Video S3 The active echo spot in the general mode.
(AVI)

Video S4 The active echo spot in the resolution mode.
(AVI)

Video S5 The active echo spot in the penetration mode.
(AVI)

Video S6 A virtual “JHU” logo is injected into the B-mode image by a single active element on the catheter tip. The experiment is performed in a water tank.
(MP4)

Video S7 Using virtual pattern injection method to indicate the ultrasound mid-plane. The experiment is performed in a water tank.
(MP4)

Video S8 Using virtual pattern injection method to indicate the ultrasound mid-plane. The experiment is performed in ex vivo chicken breast tissue.
(AVI)
